# A single mutation in the *ACTR8* gene associated with lineage-specific expression in primates

**DOI:** 10.1186/s12862-020-01620-9

**Published:** 2020-06-05

**Authors:** Se-Hee Choe, Sang-Je Park, Hyeon-Mu Cho, Hye-Ri Park, Ja-Rang Lee, Young-Hyun Kim, Jae-Won Huh

**Affiliations:** 1 National Primate Research Center, Korea Research Institute of Bioscience and Biotechnology (KRIBB), Cheongju, 28116 Korea; 2 Department of Functional Genomics, KRIBB School of Bioscience, Korea University of Science & Technology (UST), Daejeon, 34113 Korea; 3 Primate Resource Center, Korea Research Institute of Bioscience and Biotechnology (KRIBB), Jeongeup, 56216 Korea

**Keywords:** Alternative splicing, *Alu*, *ACTR8*, Exonization, Lineage-specific expression

## Abstract

**Background:**

Alternative splicing (AS) generates various transcripts from a single gene and thus plays a significant role in transcriptomic diversity and proteomic complexity. *Alu* elements are primate-specific transposable elements (TEs) and can provide a donor or acceptor site for AS. In a study on TE-mediated AS, we recently identified a novel *Alu*Sz6-exonized *ACTR8* transcript of the crab-eating monkey (*Macaca fascicularis*). In the present study, we sought to determine the molecular mechanism of *Alu*Sz6 exonization of the *ACTR8* gene and investigate its evolutionary and functional consequences in the crab-eating monkey.

**Results:**

We performed RT-PCR and genomic PCR to analyze *Alu*Sz6 exonization in the *ACTR8* gene and the expression of the *Alu*Sz6-exonized transcript in nine primate samples, including prosimians, New world monkeys, Old world monkeys, and hominoids. *Alu*Sz6 integration was estimated to have occurred before the divergence of simians and prosimians. The *Alu*-exonized transcript obtained by AS was lineage-specific and expressed only in Old world monkeys and apes, and humans. This lineage-specific expression was caused by a single G duplication in *Alu*Sz6, which provides a new canonical 5′ splicing site. We further identified other alternative transcripts that were unaffected by the *Alu*Sz6 insertion. Finally, we observed that the alternative transcripts were transcribed into new isoforms with C-terminus deletion, and in silico analysis showed that these isoforms do not have a destructive function.

**Conclusions:**

The single G duplication in the TE sequence is the source of TE exonization and AS, and this mutation may suffer a different fate of *ACTR8* gene expression during primate evolution.

## Background

Alternative splicing (AS) is a molecular mechanism producing various transcripts and diverse proteins from a single gene and plays an important role in genomic and phenotypic complexity [1, 2]. In the human genome, more than 95% of pre-mRNAs undergo AS [3, 4]. AS mechanisms are classified into five types, including exon skipping, alternative 3′ splice site (SS), alternative 5′ SS, intron retention, and mutual exclusion [5, 6]. Exon creation by duplication and mutation within repetitive elements has been reported to be highly relevant to AS and to impact mammalian evolution [7–10]. A transcriptome study of the crab-eating monkey (*Macaca fascicularis*) reported that 10% of AS events were associated with TEs [11].

TEs are mobile genetic elements that can change their position in the genome and thus can affect the sequence and structure of genes. Accordingly, they can modulate gene functions in a relatively short time and are considered as an evolutionary driving force [12, 13]. They constitute over 45% of the human genome and are categorized into two classes. Class I TEs, known as retrotransposons, require reverse transcription for their activation, and class II TEs of DNA transposons encode transposases responsible for their excision and insertion [14–16]. Retrotransposons comprise approximately 42% of the human genome and include endogenous retroviruses (ERVs); long interspersed elements (LINEs); short interspersed elements (SINEs), as well as SINE-R; variable number of tandem repeats (VNTRs); and *Alu* elements (known as SVA elements for SINE/VNTR/*Alu*) [17, 18]. *Alu* and SINE elements are the most successful of all TEs in the primate genome. *Alu* elements are slightly less than 300 base pairs (bp) in length, present at over than 1 million copies, and widely dispersed within introns and genes in the human genome [19]. *Alu* elements generally consist of distinct monomeric left and right arms, an A-rich linker, and a poly(A) tail [19]. These features facilitate the regulation of gene expression by alternative polyadenylation and non-allelic *Alu*/*Alu* recombination, causing genomic instability and eventually contributing to primate genome divergence [20–22]. In addition, when the antisense-oriented *Alu* integrates into a gene, the poly(A) tails could serve as a polypyrimidine tract (PPT) for the recognition of the cryptic splice site. Thus, *Alu* elements are a major source of cryptic exons through exonization [23]. *Alu* elements affect gene function through the generation of different splicing isoforms. Consequently, *Alu* exonization contributes to novel functions and genome evolution in primates [20, 24].

Arp8 actin-related protein 8 homolog (ACTR8) is a member of the actin superfamily and has 15–72% sequence identity with actin, which is structurally and evolutionarily similar [25]. As actin-related proteins (Arps) contain the ATP-binding pocket, termed the actin fold, their functions are distinct from those of actin [26]. Unlike actin, Arps are predominantly located in the nucleus and have been associated with nucleosome remodeling, histone acetylation, histone variant exchange, transcription regulation, and DNA repair [27–29]. ACTR8 is a key component of the INO80 complex, which has critical functions in DNA replication, repair, and recombination, as well as in transcription and heterochromatin maintenance [30, 31]. ACTR8 is involved in the ATPase activity of the INO80 complex and recruits the complex to DNA damage sites, and mutation or deletion of ACTR8 has effects similar to INO80 deletion [32]. Furthermore, ACTR8 is involved in transcriptional activation by regulating promoter architecture and contributes to the regulation of cellular function via cytoskeleton organization [33]. Despite numerous functional studies on ACTR8, information on the alternative transcripts and evolution of the *ACTR8* gene is limited.

In a study on TE-mediated AS, we recently identified a novel *Alu*Sz6-exonized *ACTR8* transcript in the crab-eating monkey [11]. Therefore, this study aimed to present a series of events of *Alu*Sz6 insertion within the *ACTR8* gene in the crab-eating monkey and explain the roles of *Alu* elements in primate evolution.

## Results

### Structural analysis of the *ACTR8* gene in various primates

**Fig. 1:**
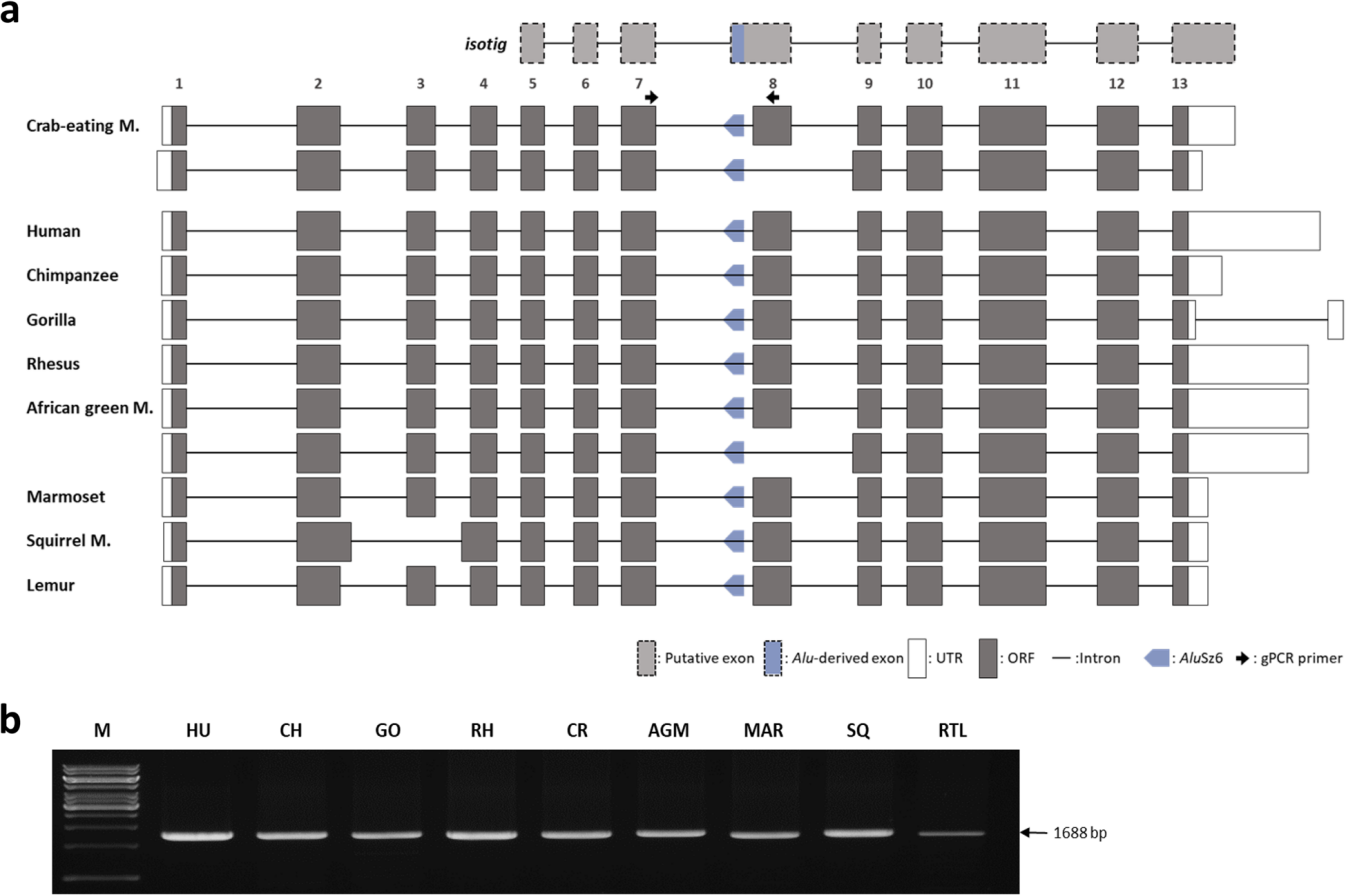
Structural analysis of the *ACTR8* gene and *Alu*Sz6 integration analysis in various primates. **a** Isotig is a putative transcript from the transcriptome study of the crab-eating monkey. Dotted boxes represent the putative exon and the *Alu*-derived exon. Open and closed boxes represent the UTR region and ORF region, respectively. In all primates, the antisense-oriented *Alu*Sz6 element is located between exon 7 and exon 8 of the *ACTR8* gene. Thick arrows indicate the location of the genomic PCR primer. This figure is schematic and not to scale. **b** Genomic PCR amplification for *Alu*Sz6 integration analysis in the *ACTR8* gene using various primate samples. M indicates the size marker. Primate DNA samples are abbreviated as follows: HU: human (*Homo sapiens*); CH: chimpanzee (*Pan troglodytes*); GO: gorilla (*Gorilla gorilla*); RH: rhesus monkey *(Macaca mulatta*); CR: crab-eating monkey (*Macaca fascicularis*); AGM: African green monkey (*Chlorocebus aethiops*); MAR: Marmoset (*Callithrix jacchus*); SQ: squirrel monkey (*Saimiri sciureus*); RTL: ring-tailed lemur (*Lemur catta*)

We conducted a structural analysis of the *ACTR8* gene in nine primates, including hominoids (human, chimpanzee, and gorilla), Old world monkeys (rhesus monkey, crab-eating monkey, and African green monkey), New world monkeys (marmoset and squirrel monkey), and prosimians (ring-tailed lemur), using DNA and mRNA sequences from the NCBI genome database. Obtained DNA sequences were screened for repetitive elements using the RepeatMasker Program, which revealed that *Alu*Sz6 is located in the 7th intron region in antisense orientation (Fig. 1a). The *ACTR8* gene is composed of 13 exons. The length of the untranslated region (UTR) differs between species, but the open reading frame (ORF) region is highly conserved and encodes 624 amino acids. Remarkably, the squirrel monkey *ACTR8* gene has 12 exons and encodes a short protein of 616 amino acids. *Alu*Sz6-exonized transcripts were not found from the NCBI database throughout the nine primates. Next, we performed genomic PCR to determine the integration time of *Alu*Sz6 using genomic DNA samples. Amplicons containing *Alu*Sz6 were detected in all the primates studied (Fig. 1b). These results indicated that *Alu*Sz6 was integrated into the primate genome before the divergence of simian and prosimian lineages.

### Identification of the *Alu*-derived and alternative splicing variants of the *ACTR8* gene

**Fig. 2:**
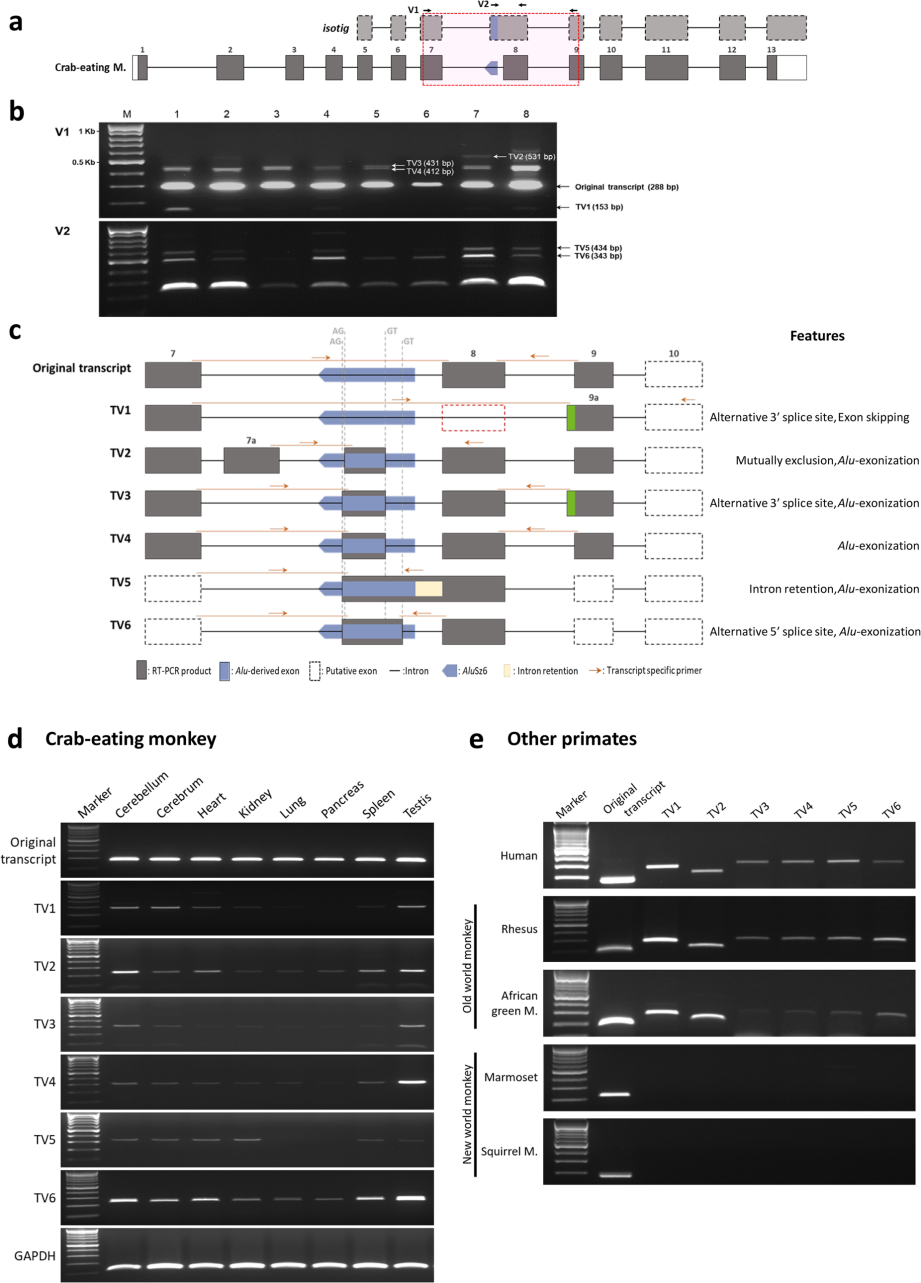
*ACTR8* gene transcript variants in primates using RT-PCR and sequence analysis. **a** The *Alu*Sz6 element-integrated transcript was validated using the V1 and V2 primer pairs. Grey and dotted boxes represent the coding exon and putative exon, respectively. Horizontal arrows indicate the V1 and V2 primer location. **b** RT-PCR was performed using the tissue cDNA of the crab-eating monkey, and the PCR product was electrophoresed by agarose gel electrophoresis and stained with ethidium bromide. Seven positive bands were observed: 1, cerebellum; 2, cerebrum; 3, heart; 4, kidney; 5, lung; 6, pancreas; 7, spleen; and 8, testis **c** Seven transcript variants were identified. Grey boxes represent the coding exon and PCR product. Vertical dashed lines represent the 3′(AG) and 5′(GT) splicing sites. The green box indicates the exon 9a, which is longer than exon 9. The orange-colored arrows represent the validation of RT-PCR primers of each transcript variant. **d** In the crab-eating monkey, RT-PCR was performed using various tissues and specific primer pairs. GAPDH (120 bp) indicates the positive control. **e** Human, Old world monkey (rhesus monkey, African green monkey), and New world monkey (marmoset, squirrel monkey) cerebellar cDNA were used for the validation of the transcript variant expression

To confirm the occurrence of *Alu*Sz6 exonization of the *ACTR8* gene in the crab-eating monkey, reverse transcription (RT) PCR was performed using two validation primer pairs (V1 and V2) (Fig. 2a and Additional file 1: Table 1). The V1 primer pairs were designed to identify transcript variants, and the V2 primers pairs were used to detect the transcripts containing the *Alu*-derived exon. In total, seven transcripts were identified in the crab-eating monkey; the V1 primers detected five transcripts, and the V2 primers detected two transcripts (Fig. 2b). Sequence analysis of the transcripts revealed that the variants originated from multiple AS events, including exon skipping, alternative 3′ SS and 5′ SS, intron retention, mutual exclusion, and *Alu* exonization (Fig. 2c) [5, 6]. The TV1 transcript skips exon 8 and has exon 9a, which is 19 bp longer than exon 9, through the use of alternative 3′ SS. The TV2 transcript has exon 7a and an *Alu*Sz6-derived exon, which are generated by mutual exclusion and *Alu* exonization, respectively. TV3 and TV4 have the same *Alu*Sz6-derived exon but carry exon 9 and exon 9a, respectively, through differential alternative 3′ SS. TV5 is generated by simultaneous *Alu*Sz6 exonization and intron retention. TV6 has a longer *Alu*Sz6-derived exon due to a differential alternative 5′ SS.

Because *Alu*-exonized transcripts often exhibit tissue-specific expression patterns [34–36], we profiled *ACTR8* gene expression in various tissues of the crab-eating monkey, including the cerebellum, cerebrum, heart, kidney, lung, pancreas, spleen, and testis. Specific RT-PCR primers for seven transcript variants were designed, considering the splice junctions of each (Fig. 2c). RT-PCR results did not reveal tissue-specific *ACTR8* gene expression. The original transcript was ubiquitously expressed in all tissues evaluated, whereas the other transcript variants (TV1–TV6) showed low or no expression in various tissues overall (Fig. 2d). We further investigated *ACTR8* gene expression of other primates (humans, rhesus monkey, African green monkey, marmoset, and squirrel monkey) using cerebellum cDNA samples and transcript variant-specific primers (Fig. 2e). In humans and Old world monkeys, transcript variants showed the same expression patterns as those of the crab-eating monkey. Moreover, each transcript variant appeared to have a similar expression level to that of the crab-eating monkey. Remarkably, New world monkeys only expressed the original transcript. Our data suggest that the expression of the *ACTR8* gene is regulated by lineage-specific AS events in primates.

### Bioinformatics analysis of alternative splicing transcripts

**Fig. 3:**
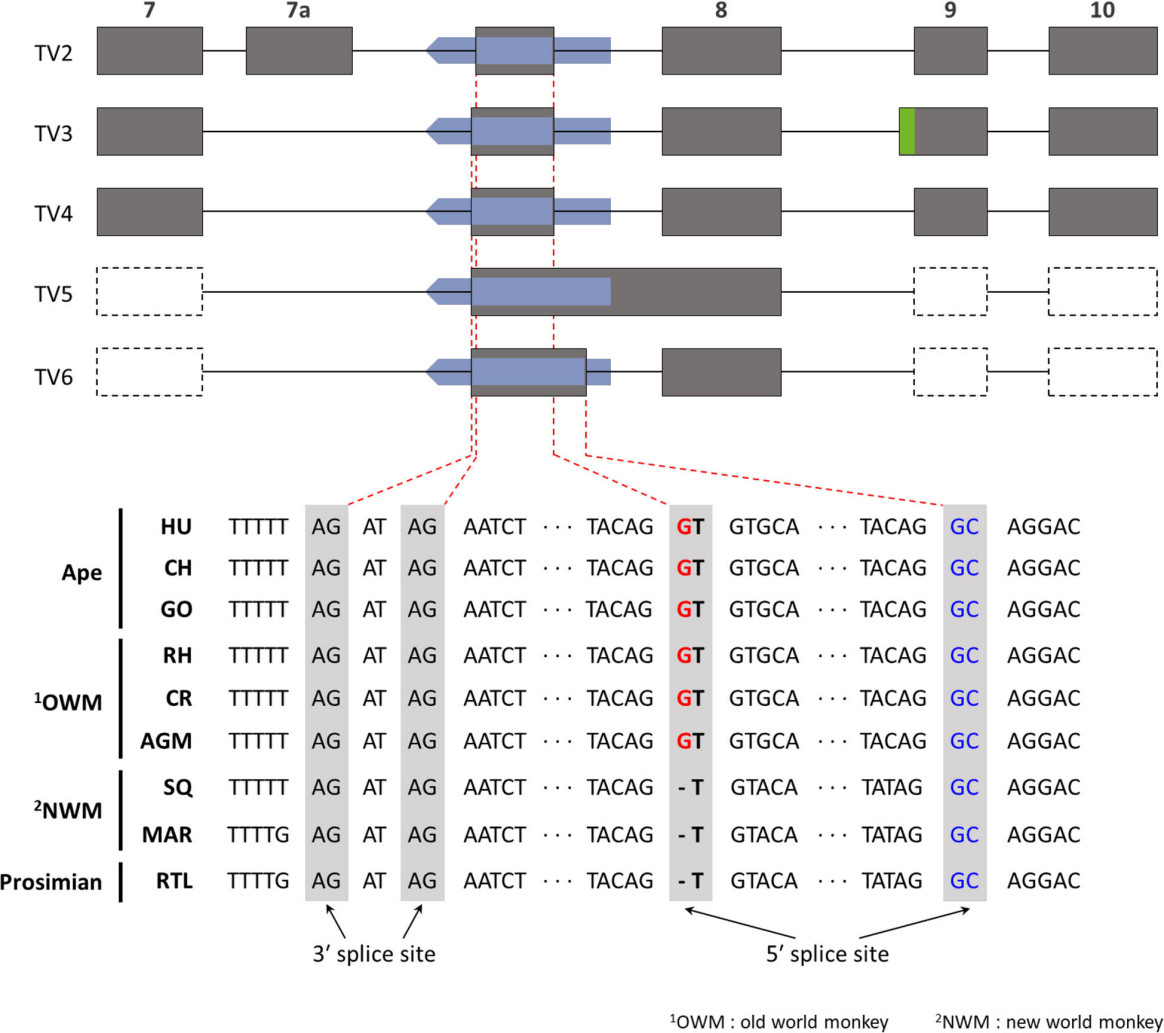
*Alu*Sz6 integration analysis in the *ACTR8* gene in various primates. Boundary sequence of potential 3′ and 5′ splice sites in various primates. Grey boxes indicate the splice site by the *Alu*Sz6 element. Primates are abbreviated as follows: HU: human (*Homo sapiens*); CH: chimpanzee (*Pan troglodytes*); GO: gorilla (*Gorilla gorilla*); RH: rhesus monkey (*Macaca mulatta*); CR: crab-eating monkey (*Macaca fascicularis*); AGM: African green monkey (*Chlorocebus aethiops*); MAR: Marmoset (*Callithrix jacchus*); SQ: squirrel monkey (*Saimiri sciureus*); RTL: ring-tailed lemur *(Lemur catta*)

Multiple sequence alignment of the *Alu*Sz6-derived exon in nine primates (humans, chimpanzee, gorilla, rhesus monkey, African green monkey, marmoset, squirrel monkey, and ring-tailed lemur) demonstrated that a novel G duplication was used as the new 5′ SS for the *Alu*Sz6-derived exon in Old world monkeys and apes (Fig. 3 and Additional file 1: Figure S1A). Considering our results, lineage-specific *Alu* exonization in the *ACTR8* gene might be caused by the G duplication mutation, which is absent in New world monkeys and prosimians. We carefully proved our findings that difference *Alu*Sz6 sequences among the primates, matching with *Alu*Sz6 sequences from the UCSC genome browser. We found a discrepant G duplication in the *Alu*Sz6 sequence in primate lineages (Additional file 1: Figure S4).

Moreover, we identified lineage-specific exon inclusion or exclusion events, regardless of *Alu*Sz6 integration. The TV2 transcript carrying exon 7a showed lineage-specific expression. The splice sites (donor and acceptor site) were well conserved in all primates evaluated (Additional file 1: Figure S1). Assessment of the branch point using the SVM-BP finder to the surrounding sequences of exon 7a predicted several branch point candidates. Among the candidates, the TTATAAGAT sequence had the highest potential for inclusion in exon 7a. This sequence was located 21 bp upstream of the 3′ SS of exon 7a in Old world monkeys and apes, but not New world monkeys and prosimians (Additional file 1: Figure S1). It is likely that a lineage-specific mutual exclusion exon, exon 7a, may have been spliced due to the branch point difference (Additional file 1: Figure S1). In the squirrel monkey, exons 2 and 3 were found to be longer than in the other primates (Fig. 1a). We examined the splice site of the constitutive exon. Interestingly, the squirrel monkey has a TA sequence, a specific acceptor splice site, whereas Old world monkeys and apes do not have the 5′ SS consensus sequence in the same region (Additional file 1: Figure S2). The TA acceptor splice site is also seen in marmosets and lemurs, and although they are likely to express the longer exons 2 and 3, we did not confirm their expression experimentally (Additional file 1: Figure S2). Therefore, besides *Alu*Sz6, factors promote lineage-specific expression in the primate *ACTR8* gene.

### Analysis of ACTR8 isoform structure and function

**Fig. 4:**
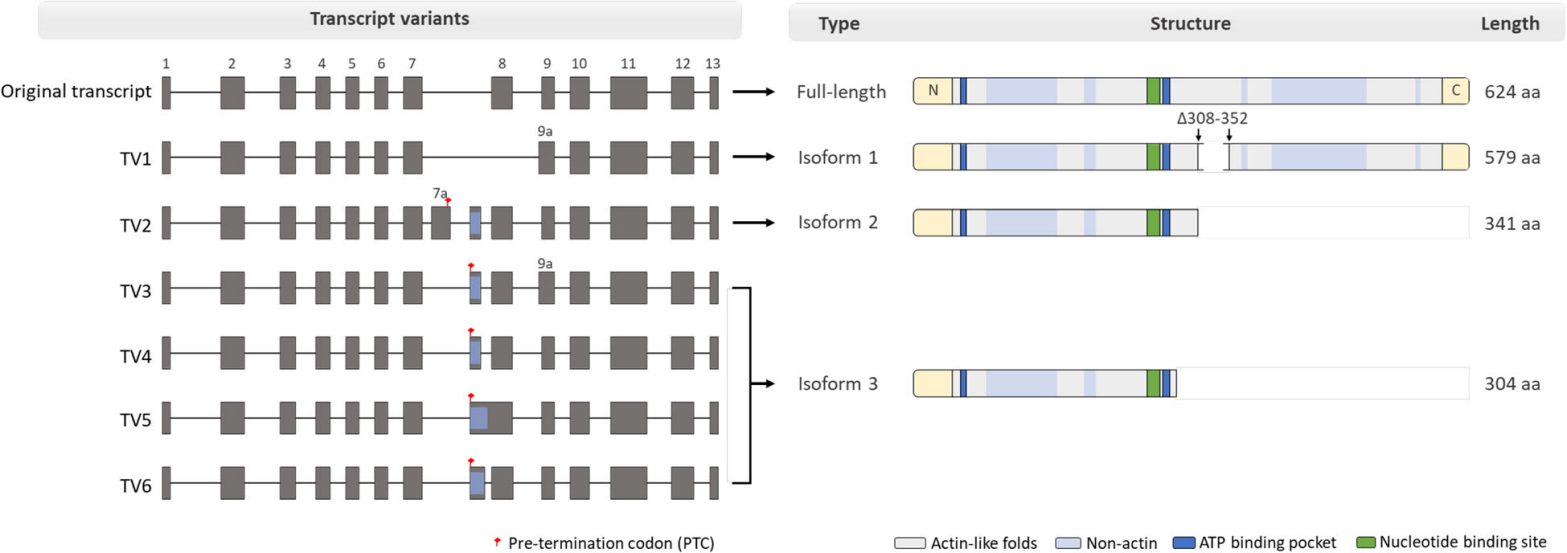
Schematic of the genomic and protein structures of the *ACTR8* gene. Full-length *ACTR8* of humans and crab-eating monkeys encoding 624 amino acids containing the actin, ATP-binding, and nucleotide-binding sites. The identified transcript variants encoded isoform 1 and isoforms 2–3 lacking the C-terminal

To assess how the seven transcript variants identified affect translation and protein function, we performed in silico analysis using the ORF finder (http://www.ncbi.nlm.nih.gov/projects/gorf/) and Pfam (https://pfam.xfam.org) database. In this study, seven transcript variants produced a total of four isoforms of the ACTR8 protein, containing the original and alternative isoforms. The original transcript encodes the full-length protein with 624 amino acids, including an ATP-binding site (amino acids (aa) 55–56, 288, and 290) and a nucleotide-binding site (aa 283–286) [37, 38]. The TV1 transcript encodes isoform 1 with 579 aa, deleting aa 308 to 352. TV2 encodes isoform 2 with 341 aa, and TV3–TV6 encode isoform 3 with 304 aa (Fig. 4). Exon 7a and *Alu*Sz6 introduce a pre-termination codon (PTC) in TV2–TV6, and we identified truncated proteins, with isoforms 2 and 3 deleted from the C-terminus (Additional file 1: Figure S3). Notably, isoforms 2 and 3 certainly contained the ATP-binding pocket and nucleotide-binding sites, which are essential functional domains for correct ACTR8 functioning. According to a previous study, the N-terminal region of ACTR8 is critical for protein functional activity. N-terminal deletions have deleterious effects on the expressed protein [39–41]. However, deletions in the C-terminal region did not lead to defects in ACTR8 function [37]. Although the *Alu*-derived transcript in the *ACTR8* gene altered the protein structure, obtained novel isoforms can produce a lineage-specific protein without loss of function.

## Discussion

*Alu* elements belong to the SINE family of TEs and are primate-specific repetitive elements. In humans, they make up 11% of the genome. *Alu* elements emerged 65 million years ago, before the radiation of primates, by a fusion of the 5′ and 3′ ends of the 7 SL RNA gene [42, 43]. Once an *Alu* integrated at a chromosome locus, it might have copied itself for transposition. Thus, its copy numbers increased throughout primate evolution [19, 44]. Although *Alu* elements inserted in non-coding regions are thought to be evolutionarily neutral, many scientists have demonstrated that TEs evidently play a key role in primate evolution by creating new mutations and gene combinations [45]. *Alu* elements integrated into the human genome sometimes have little effect on the phenotype [43], but *Alu* elements created a significant increase in transcriptome diversity and contributed to unique features of primates [19, 44, 45]. The integration time of locus-specific *Alu* elements can indicate evolutionary time in primate lineages. *Alu* elements are useful tools to study genome evolution, phylogenetics, and population biology in primates [45, 46]. Moreover, *Alu*-exonized transcripts can be useful for analyzing the mechanism underlying the creation of new transcript variants during primate evolution by estimating the transcript generation time [47–49].

*Alu* elements inserted in the human genome are distributed to introns in both 55% antisense and 45% sense orientations [50]. The sense-orientation *Alu* elements provide splicing silencer sequences that repress their exonization [51], while antisense oriented *Alu*-elements contain strong PPT and many potential 5′ and 3′ splice sites and therefore tend to be alternatively spliced at a higher frequency than the original exon [24, 50]. *Alu* elements can be divided into distinct lineages or families based on inheritance patterns of new mutations [52]. The *Alu*S subfamily, to which *Alu*Sz6 belongs, is the second oldest *Alu* lineage and ~ 30 million years old, and it comprises 551,383 full-length copies in humans [53]. The *Alu*S subfamily has recently been reported to be active in humans [54], and editing events such as A to I RNA editing occurs with high frequency in this subfamily [55]. Furthermore, *Alu*-mediated unequal recombination has been reported, resulting in mutation or polymorphic substitution, implying contribution to structure variation and *Alu* evolution [56, 57]. In addition, insertion of *Alu* elements might cause genetic disease and cancer through structural variation and genomic instability [58–61]. In fact, partial *Alu*Sz6 insertion in the *ACAT1* gene has a negative effect downstream of exon inclusion, depending on the distance between the *Alu* element and the splice site acceptor site [62].

This study aimed to evaluate the *Alu*Sz6 integration event in the *ACTR8* gene. Here, we discovered for the first time that *Alu*Sz6 has existed in a dormant state as junk DNA in the *ACTR8* gene, and it was activated in the *ACTR8* gene in Old world monkey and ape lineages by a small mutation: G duplication (Fig. 3). We tried to rationalize our result that lineage-specific expression is caused by G duplication in *Alu*Sz6. First, we validated our results based on findings from compiled data about *Alu* exonization in humans [63]. *Lev-Maor* et al. showed that an inverted *Alu* insertion sequence used a 3′ SS, proximal AG, and this is especially well shown in the *Alu*S subfamily. Our results indicated that the use of proximal AG was preferred over distal AG, similar to the findings of *Lev-Maor* et al. [63]. Second, we suggest the molecular mechanism of the G duplication event in the *Alu*Sz6 element. Two molecular mechanisms might explain our results: there might be secondary unequal homologous recombination or DNA replication-derived errors [60, 64, 65]. Commonly, unequal homologous recombination is characterized by nearby *Alu* elements or L1 sequence in the genome and duplication or deletions of the segments [66]. We speculated that the single G duplication in *Alu*Sz6 is the error that occurred during DNA replication. Therefore, we found that molecular mechanism about the occurrence of single mutation in *Alu*Sz6.

The original transcript (288 bp) was more strongly expressed compared to the other transcripts (153 bp, 412 bp, 431 bp, and 531 bp). The original transcript showed a 13.1-fold higher intensity compared to the weakly expressed transcript TV4 (Additional file 1: Figure S5). These gaps in expression levels suggest that the original transcript is a constitutive transcript, and that the other transcript variants are the minor expressed forms. Moreover, in various tissues, other transcript variants except the original were either not expressed or very weakly expressed (Fig. 2d, e).

**Fig. 5:**
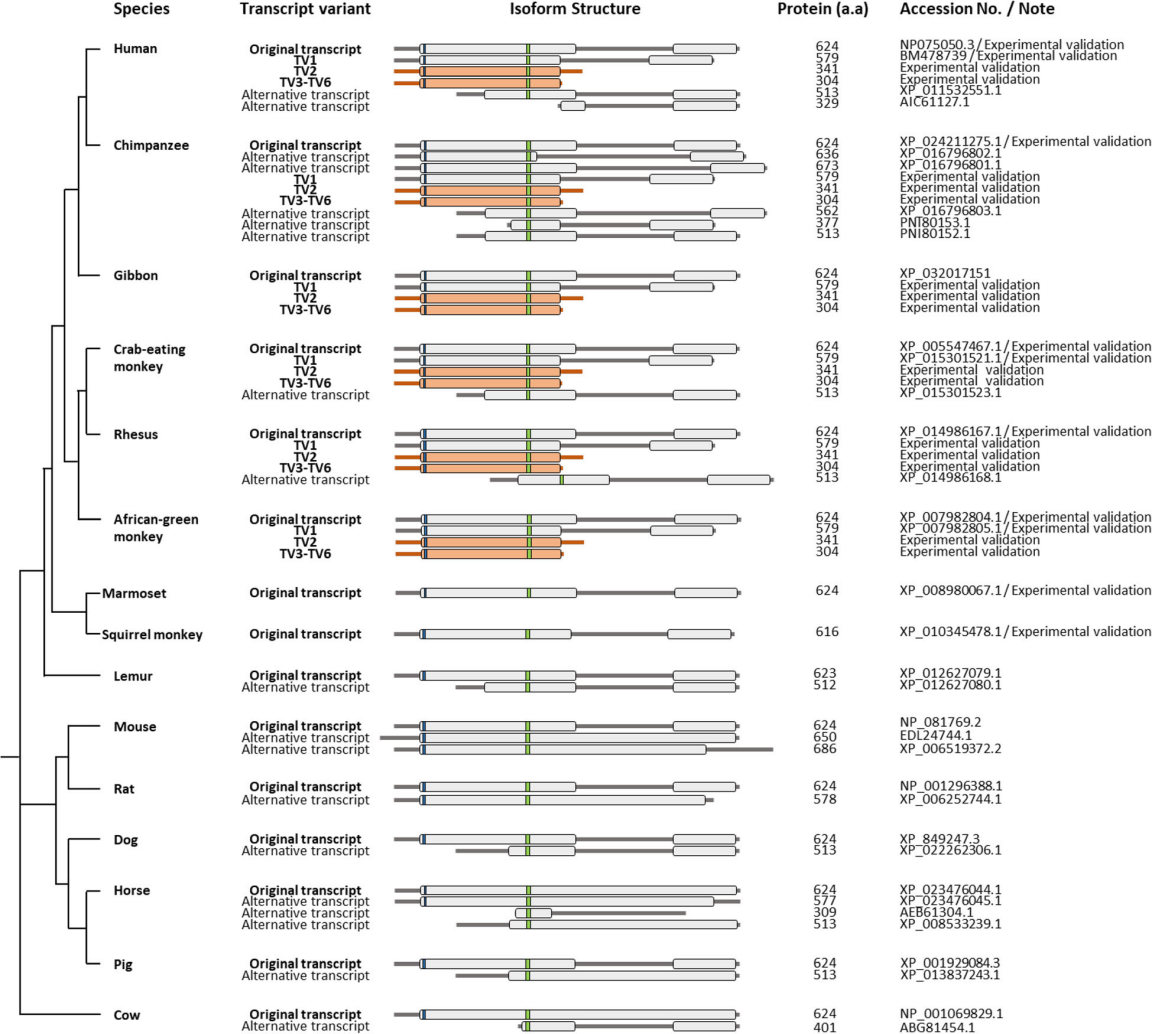
Schematic representation of the experimental methods and in silico analysis of the ACTR8 isoform in primates, mouse, rat, dog, horse, cow, and pig. Phylogenetic analysis of the full-length ACTR8 isoform in mammals. Narrow blue and green rectangles indicate the ATP-binding pocket and nucleotide-binding site, respectively. Orange represents the truncated isoform with C-terminal deletion

ACTR8 links to H2A via the C-terminal, and the N-terminal binds to the DNA. Recent studies have reported that C-terminal loss does not affect functional activity [37, 41]. Our newly identified transcript variants express a truncated protein with the deletion of the C-terminal-truncated yet functional protein, containing the two core domain sequences (Fig. 4). Thus, we posit that the novel ACTR8 isoforms caused by *Alu*Sz6 insertion do not lead to functional defects in the protein. There are only lineage-specific differences in primates. To understand the lineage-specific evolution of ACTR8, we identified and analyzed ACTR8 isoforms in some primates, as well as representative mammals (mouse, rat, dog, horse, cow, and pig) (Fig. 5). Schematic comparison among the 15 mammals was performed on the basis of registered protein information in NCBI resources. Furthermore, the results of the experimental validation from primates were added to the protein comparison sets. As expected, full-length isoforms were highly conserved in all tested mammals with 624 amino acids. Intriguingly, apes and Old world monkeys expressed isoforms comprising the skipping transcript (TV1) and C-terminal deletion isoform by *Alu*Sz6 integration. Besides, N-terminal-deleted isoforms were registered in the protein database of NCBI for human, chimpanzee, crab-eating monkey, rhesus monkey, lemur, dog, pig, and cow. Nevertheless, apes and Old world monkeys were able to generate truncated isoforms without being affected by the loss of ACTR8 function. Non-primate mammals did not produce the C-terminal deletion isoform owing to the absence of the integration of TE, particularly primate-specific TE. There is an appearance of primate-specific ACTR8 proteins. Consequently, these results were meaningful in that primates, including humans, may just get a chance for genomic diversity enhancement in a way that only primates could do through *Alu* element.

**Fig. 6:**
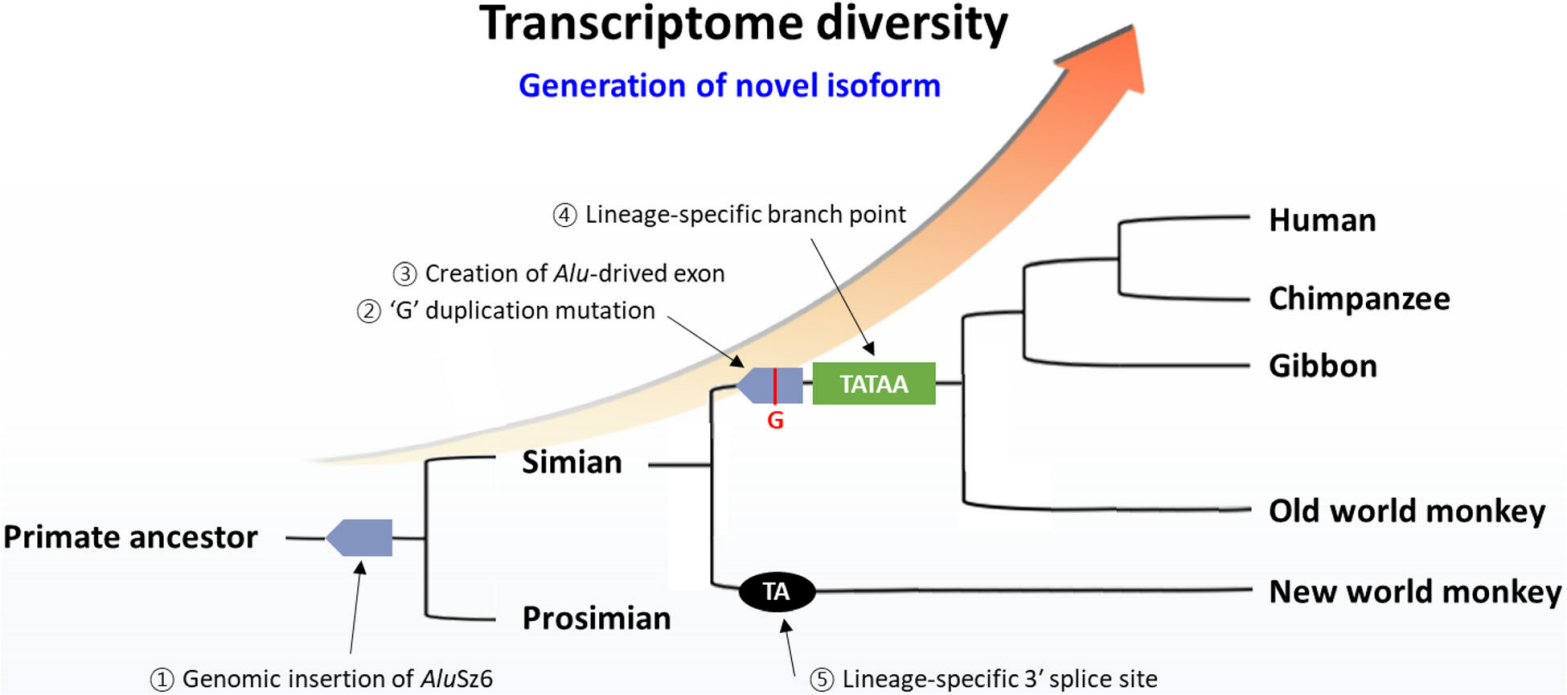
Schematic representation of the evolutionary events in primate *ACTR8* genes. Events during the primate evolution of the *ACTR8* gene are summarized

Taken together, our findings indicate that the *Alu*Sz6 integration event occurred prior to primate radiation. Also, the single G duplication in *Alu*Sz6 provided mechanism to production of the *Alu*-derived exon and presented in *ACTR8* gene of apes and Old world monkeys (Fig. 6). At the same time, Old world monkeys and apes acquired a lineage-specific branch-point of the TATATAAGAT sequence. In addition, we characterized the unique splice donor site in New world monkeys following the lineage-specific transcript. Therefore, not only a single duplication in *Alu*Sz6, but also other lineage-specific genomic features appear to be concerned with lineage-specific expression and transcriptomic diversity, which may eventually play an important role in primate evolution.

## Conclusions

The current study revealed the step-by-step evolutionary events in the *ACTR8* gene that contributed to transcriptome diversity and the generation of novel isoforms of this gene in primates.

## Methods

### Total RNA and genomic DNA extraction

The total RNA samples isolated from different human tissues, including the cerebellum, cerebrum, heart, kidney, lung, liver, spleen, and testis, were purchased from Clontech Laboratories, Inc. The total RNA of non-human primates was extracted from the following tissues of the following animals: crab-eating monkey (cerebellum, cerebrum, heart, kidney, lung, pancreas, spleen, and testis), African green monkey (cerebrum), and common marmoset (cerebrum). The RNA samples were isolated using the RNeasy® Plus Mini kit (Qiagen), according to the manufacturer’s instructions. DNA contamination was eliminated by using the genomic DNA eliminator column and RNase-free DNase (Qiagen). The RNA concentration and purity (A260/A280) were determined with the ND-1000 spectrophotometer (NanoDrop Technologies), and the integrity of RNA was further confirmed by running the samples on an agarose gel. About 500 ng of total RNA was used for complementary DNA synthesis using the GoScript Reverse Transcription System (Promega) according to the manufacturer’s instructions. The primate genomic DNA samples (HU: *Homo sapiens*, CH: *Pan troglodytes*, GO: *Gorilla gorilla*, RH: *Macaca mulatta*, CR: *Macaca fascicularis*, AGM: *Cercopithecus aethiops*, MAR: *Callithrix jacchus*, SQ: *Saimiri sciureus*, RTL: *Lemur catta*) and all the non-human primate samples were provided by the National Primate Research Center (NPRC) of Korea.

### RT-PCR and genomic PCR

*ACTR8* transcripts were analyzed by RT-PCR amplification using the following primer pairs: V1 (S: 5′-AGG AGT CTG TGT GTG CCA-3′, AS: 5′-GAA GCC CAG AGA TGT CCT GA-3′); V2 (S: 5′-TTT AAG GTG TTG CAG AAG AAG AC-3′, AS: 5′-TTT AAG GTG TTG CAG AAG AAG AC-3′). RT-PCR was conducted using the ExPrime Taq premix (Genetbio) under the following conditions: pre-denaturation for 5 min; 30 ~ 35 cycles at 94 °C for 30 s, 57–61 °C for 30 s, 72 °C for 30 ~ 40 s; and a final elongation at 72 °C for 5 min. To analyze the integration time of the *Alu*Sz6 element during primate evolution, genomic DNAs from different primate lineages were amplified using specific primer sets designed from well-conserved regions (S: 5′-TAT GCT GTG TGG AGG ATG GG-3′, AS: 5′-CTG GCA TTC TCT GTA AGG GAA C-3′). The genomic PCR was carried out under the following conditions: 94 °C for 5 min; 30 cycles at 94 °C for 30 s, at 56 °C for 30 s, at 72 °C for 90 s; and a final elongation step at 72 °C for 5 min. The *GAPDH* gene was used as a standard control and was analyzed using specific primer pairs (S: 5′- GAA ATC CCA TCA CCA TCT TCC AGG-3′, AS: 5′- GAG CCC CAG CCT TCT CCA TG- 3′) designed based on the human *GAPDH* sequence.

### Molecular cloning and sequencing

For the validation and sequencing of the PCR products, all PCR products were separated on a 1.5% agarose gel, purified with the Gel SV Extraction kit (GeneAll), and cloned into the RBC T&A Cloning Vector. The cloned DNA was isolated using the Plasmid DNA Mini-prep kit (GeneAll) and sequenced by Macrogen. The validation of primate DNA samples and alternative transcripts was performed as mentioned above.

### Branch-point analysis

Splicing is catalyzed by the spliceosome, a large RNA–protein complex, and this complex binds to the branch point site, which is a short sequence upstream of the acceptor splice sites (3′ SS). As the branch point sequences are associated with the cryptic and canonical 3′ SSs, branch-point prediction is important in pre-mRNA splicing. The SVM-BPfinder program (http://regulatorygenomics.upf.edu/Software/SVM_BP/) [67] was used to analyze the branch point site in the 7th intron sequence of the *ACTR8* gene.

### Comparative in silico analysis of the ACTR8 protein in different species

All ACTR8 protein sequences were collected from the NCBI database in FASTA format for the following different species: human, chimpanzee, gibbon, crab-eating monkey, rhesus monkey, African green monkey, marmoset, rat, dog, horse, cow, and pig. The BioEdit program was used for multiple sequence comparison. Pairwise alignments were used to determine the identity and similarity between each species. On the basis of the full-length sequence of the ACTR8 protein, the phylogeny tree of the ACTR8 protein was obtained by the BioEdit and TreeView programs.

### Acquisition and quantitative analysis of PCR-gel images

The separated PCR products on the ethidium bromide-stained gels were scanned using the gel image software (Vision-capt, Vilber). Band intensity was calculated by the volume, based on the height and area of the positive bands, and the relative intensity (%) was determined.

## Supplementary information

**Additional file 1: Table S1.** List of primers used in this study. **Figure S1** Multiple sequence alignment of the *Alu*Sz6 insertion region in the *ACTR8* gene in various primates. Sequences obtained from the genomic PCR product (Fig. 1b). Black lined boxes indicate exons, and sequences between boxes are intron regions. Two forward slashes indicate the region of omitted intron sequence for good-quality data. The sky-blue region represents the full length of *Alu*Sz6. 3′ Splice sites and 5′ splice sites in the *Alu*Sz6 region are not in sky blue. The black-shaded region indicates the branch point site, and New world monkeys and prosimians have a deletion in this branch point region. **Figure S2** Multiple sequence alignment of the region from exon 2 to exon 4 in the *ACTR8* gene in various primates. Genomic sequences from the NCBI database were aligned in various primates. The donor splice site (5′ splice site) and acceptor splice site (3′ splice site) are indicated as blue and red dotted line boxes, respectively. **Figure S3** Multiple sequence alignment of the RT-PCR products using the validation primer. The RT-PCR products were sequenced, and these sequences were analyzed by multiple nucleotide alignment. The sky-blue region indicates the *Alu*Sz6-derived exon region. The red lined box represents the premature termination codon (PTC). **Figure S4** Multiple sequence alignment of *Alu*Sz6 of the *ACTR8* gene. *Alu*Sz6 sequences of various primates were searched using online resources (UCSC Genome browser). In this figure, the *Alu*Sz6 sequences are shown in the forward direction (5′ to 3′), whereas the main figure is shown in the reverse direction as inserted into the genome. The blue lined box indicates the point mentioned as a G duplication. *Alu*Sz6 of RTL could not be analyzed, but we confirmed the *Alu*Sz6 sequence of RTL through the experiment. **Figure S5** Analysis of the *ACTR8* gene PCR band intensity. (A) Green lanes (1–5) were PCR-positive bands by the V1 primer from Fig. 2b. Vision-capt software selected five bands and generated peaks of the corresponding PCR band. The PCR band intensity was generated from these peaks. (B) Vision-capt software measured the volume based on height and area of the positive bands, respectively.

## Data Availability

All figures and tables generated in this study are available in this article and its additional files.

## Abbreviations

AS: Alternative splicing
Arps: Actin-related proteins
bp: Base pairs
ERVs: Endogenous retroviruses
LINEs: Long interspersed elements
ORF: Open reading frame
PTC: Premature termination codon
SINEs: Short interspersed elements
3′ SS: 3′ splice site
TE: Transposable elements
UTR: Untranslated region
VNTRs: Variable number of tandem repeats
PPT: Polypyrimidine tract
