# Supplementary material for: A single mutation in the ACTR8 gene associated with lineage-specific expression in primates
Source: BMC Evol Biol. 2020 Jun 5;20:66. doi: 10.1186/s12862-020-01620-9 (PMC7275561; doi:10.1186/s12862-020-01620-9)
Supplement: Supplementary file 1 — Additional file 1: Table S1. List of primers used in this study. Figure S1 Multiple sequence alignment of the AluSz6 insertion region in the ACTR8 gene in various primates. Sequences obtained from the genomic PCR product (Fig. 1b). Black lined boxes indicate exons, and sequences between boxes are intron regions. Two forward slashes indicate the region of omitted intron sequence for good-quality data. The sky-blue region represents the full length of AluSz6. 3′ Splice sites and 5′ splice sites in the AluSz6 region are not in sky blue. The black-shaded region indicates the branch point site, and New world monkeys and prosimians have a deletion in this branch point region. Figure S2 Multiple sequence alignment of the region from exon 2 to exon 4 in the ACTR8 gene in various primates. Genomic sequences from the NCBI database were aligned in various primates. The donor splice site (5′ splice site) and acceptor splice site (3′ splice site) are indicated as blue and red dotted line boxes, respectively. Figure S3 Multiple sequence alignment of the RT-PCR products using the validation primer. The RT-PCR products were sequenced, and these sequences were analyzed by multiple nucleotide alignment. The sky-blue region indicates the AluSz6-derived exon region. The red lined box represents the premature termination codon (PTC). Figure S4 Multiple sequence alignment of AluSz6 of the ACTR8 gene. AluSz6 sequences of various primates were searched using online resources (UCSC Genome browser). In this figure, the AluSz6 sequences are shown in the forward direction (5′ to 3′), whereas the main figure is shown in the reverse direction as inserted into the genome. The blue lined box indicates the point mentioned as a G duplication. AluSz6 of RTL could not be analyzed, but we confirmed the AluSz6 sequence of RTL through the experiment. Figure S5 Analysis of the ACTR8 gene PCR band intensity. (A) Green lanes (1–5) were PCR-positive bands by the V1 primer from Fig. 2b. Vision-capt software s [file 12862_2020_1620_MOESM1_ESM.pptx]

## Slide 1
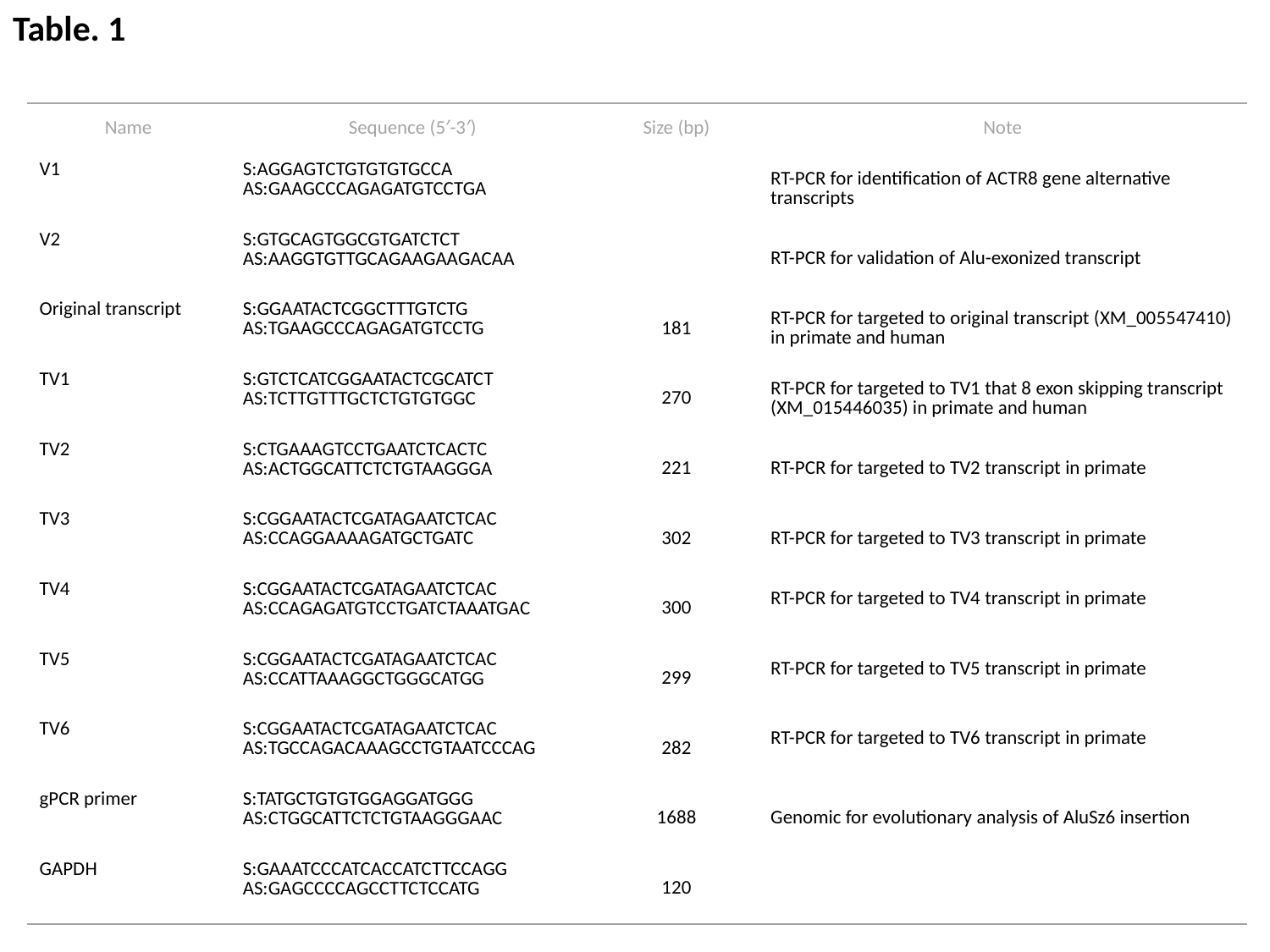

Table. 1
| Name | Sequence (5′-3′) | Size (bp) | Note |
| --- | --- | --- | --- |
| V1 | S:AGGAGTCTGTGTGTGCCA AS:GAAGCCCAGAGATGTCCTGA | | RT-PCR for identification of ACTR8 gene alternative transcripts |
| V2 | S:GTGCAGTGGCGTGATCTCT AS:AAGGTGTTGCAGAAGAAGACAA | | RT-PCR for validation of Alu-exonized transcript |
| Original transcript | S:GGAATACTCGGCTTTGTCTG AS:TGAAGCCCAGAGATGTCCTG | 181 | RT-PCR for targeted to original transcript (XM\_005547410) in primate and human |
| TV1 | S:GTCTCATCGGAATACTCGCATCT AS:TCTTGTTTGCTCTGTGTGGC | 270 | RT-PCR for targeted to TV1 that 8 exon skipping transcript (XM\_015446035) in primate and human |
| TV2 | S:CTGAAAGTCCTGAATCTCACTC AS:ACTGGCATTCTCTGTAAGGGA | 221 | RT-PCR for targeted to TV2 transcript in primate |
| TV3 | S:CGGAATACTCGATAGAATCTCAC AS:CCAGGAAAAGATGCTGATC | 302 | RT-PCR for targeted to TV3 transcript in primate |
| TV4 | S:CGGAATACTCGATAGAATCTCAC AS:CCAGAGATGTCCTGATCTAAATGAC | 300 | RT-PCR for targeted to TV4 transcript in primate |
| TV5 | S:CGGAATACTCGATAGAATCTCAC AS:CCATTAAAGGCTGGGCATGG | 299 | RT-PCR for targeted to TV5 transcript in primate |
| TV6 | S:CGGAATACTCGATAGAATCTCAC AS:TGCCAGACAAAGCCTGTAATCCCAG | 282 | RT-PCR for targeted to TV6 transcript in primate |
| gPCR primer | S:TATGCTGTGTGGAGGATGGG AS:CTGGCATTCTCTGTAAGGGAAC | 1688 | Genomic for evolutionary analysis of AluSz6 insertion |
| GAPDH | S:GAAATCCCATCACCATCTTCCAGG AS:GAGCCCCAGCCTTCTCCATG | 120 | |

## Slide 2
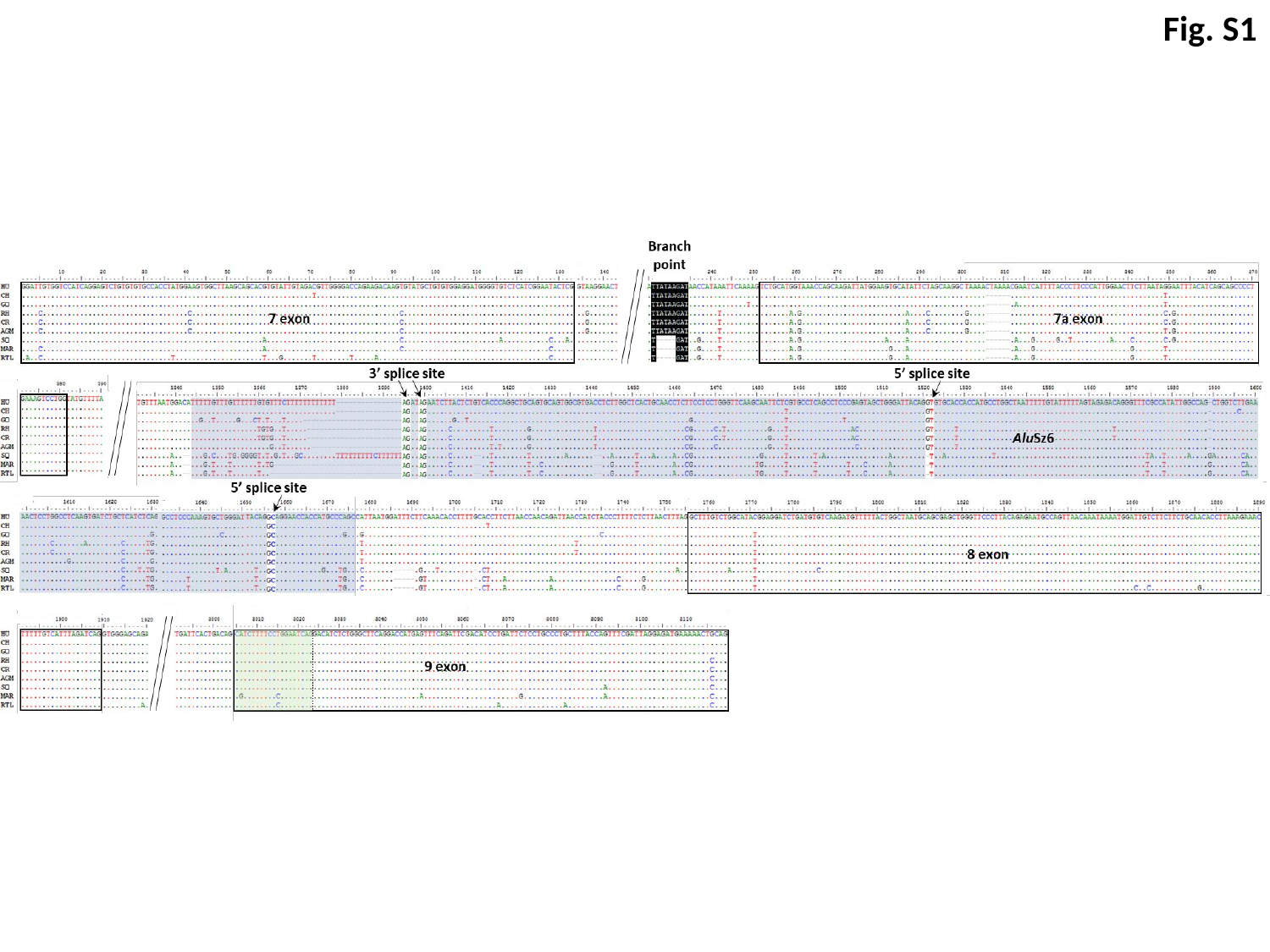

Fig. S1

## Slide 3
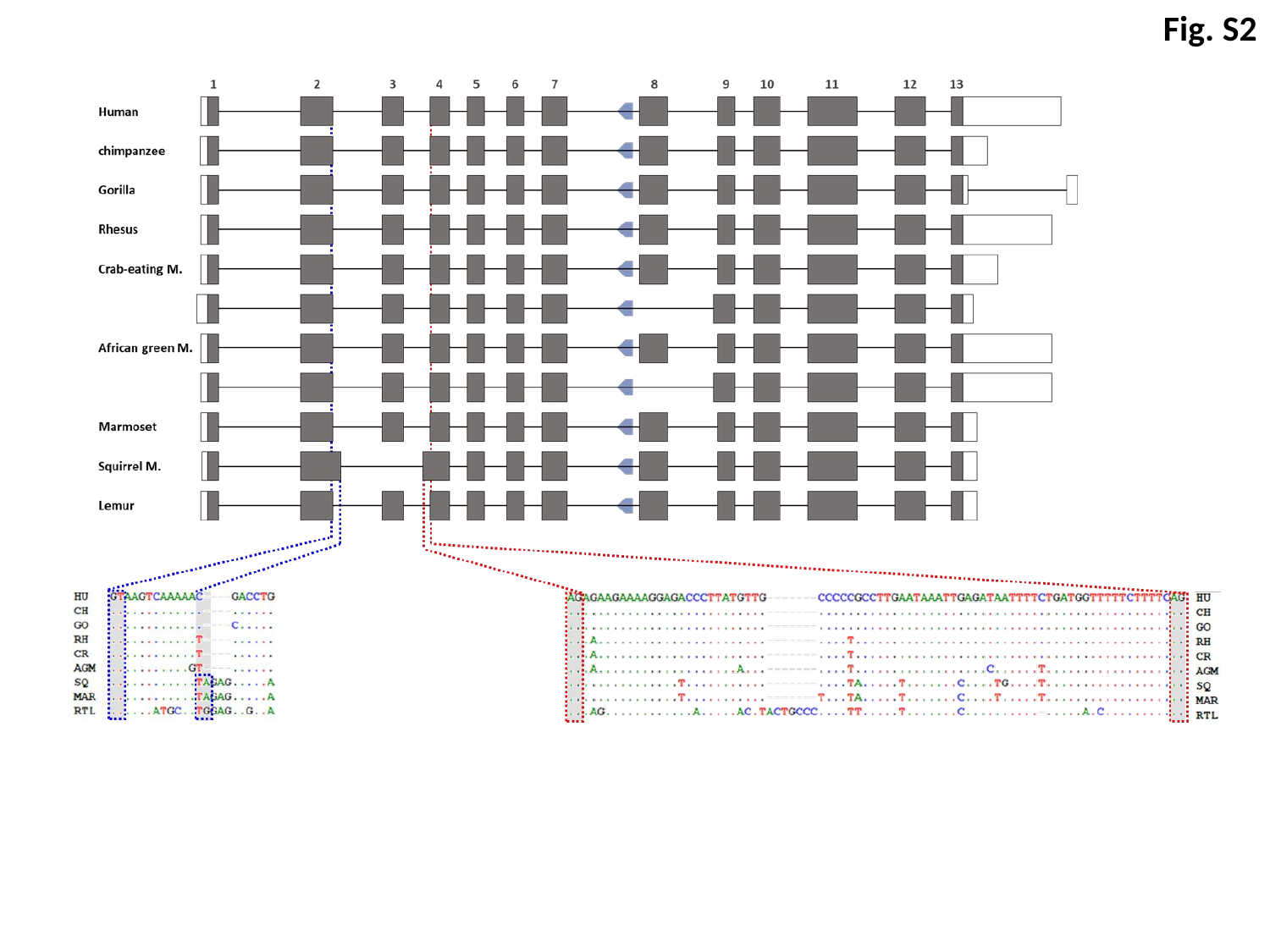

Fig. S2

## Slide 4
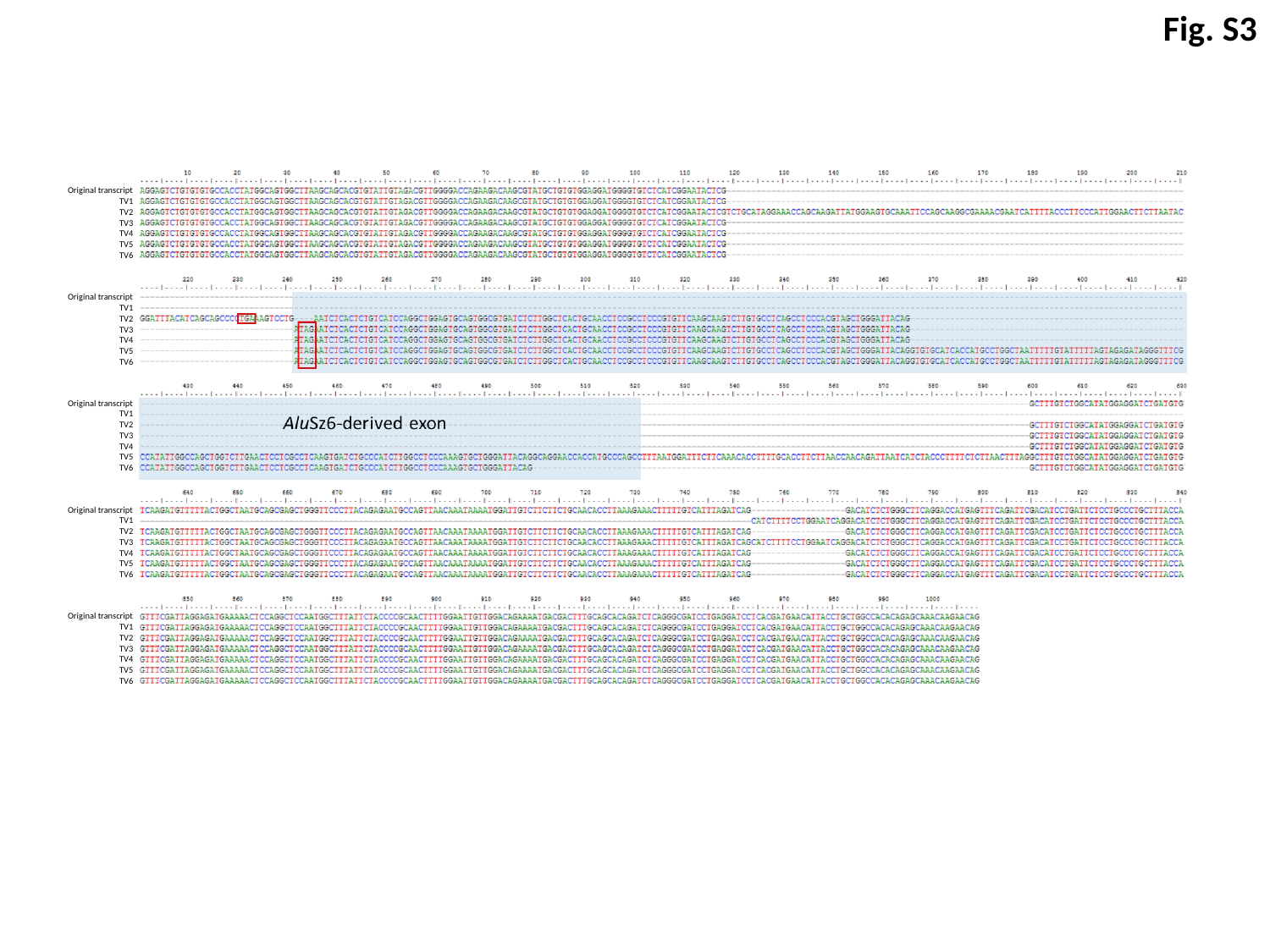

Fig. S3
Original transcript
TV1
TV2
TV3
TV4
TV5
TV6
Original transcript
TV1
TV2
TV3
TV4
TV5
TV6
Original transcript
TV1
TV2
TV3
TV4
TV5
TV6
Original transcript
TV1
TV2
TV3
TV4
TV5
TV6
Original transcript
TV1
TV2
TV3
TV4
TV5
TV6

## Slide 5
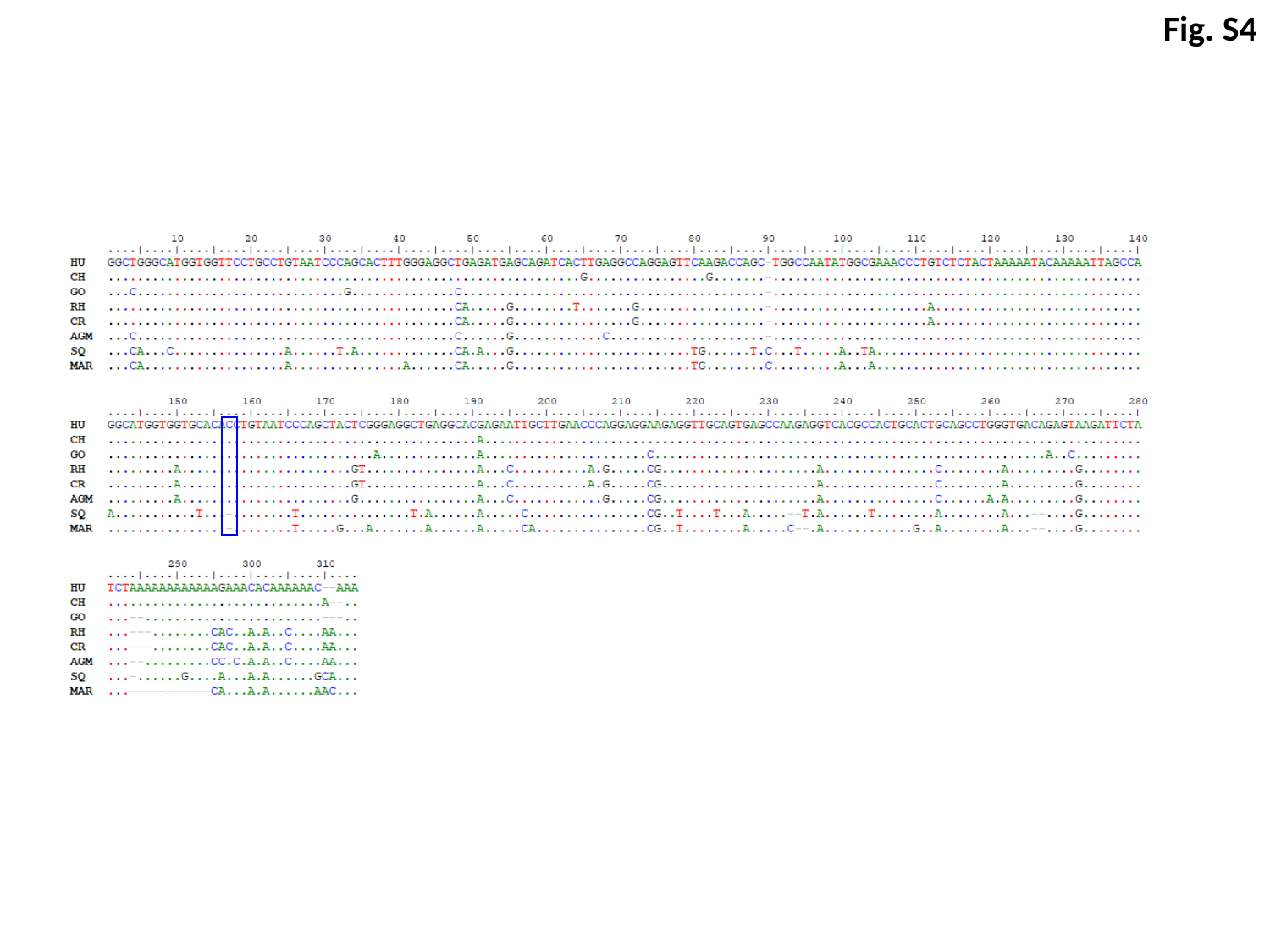

Fig. S4

## Slide 6
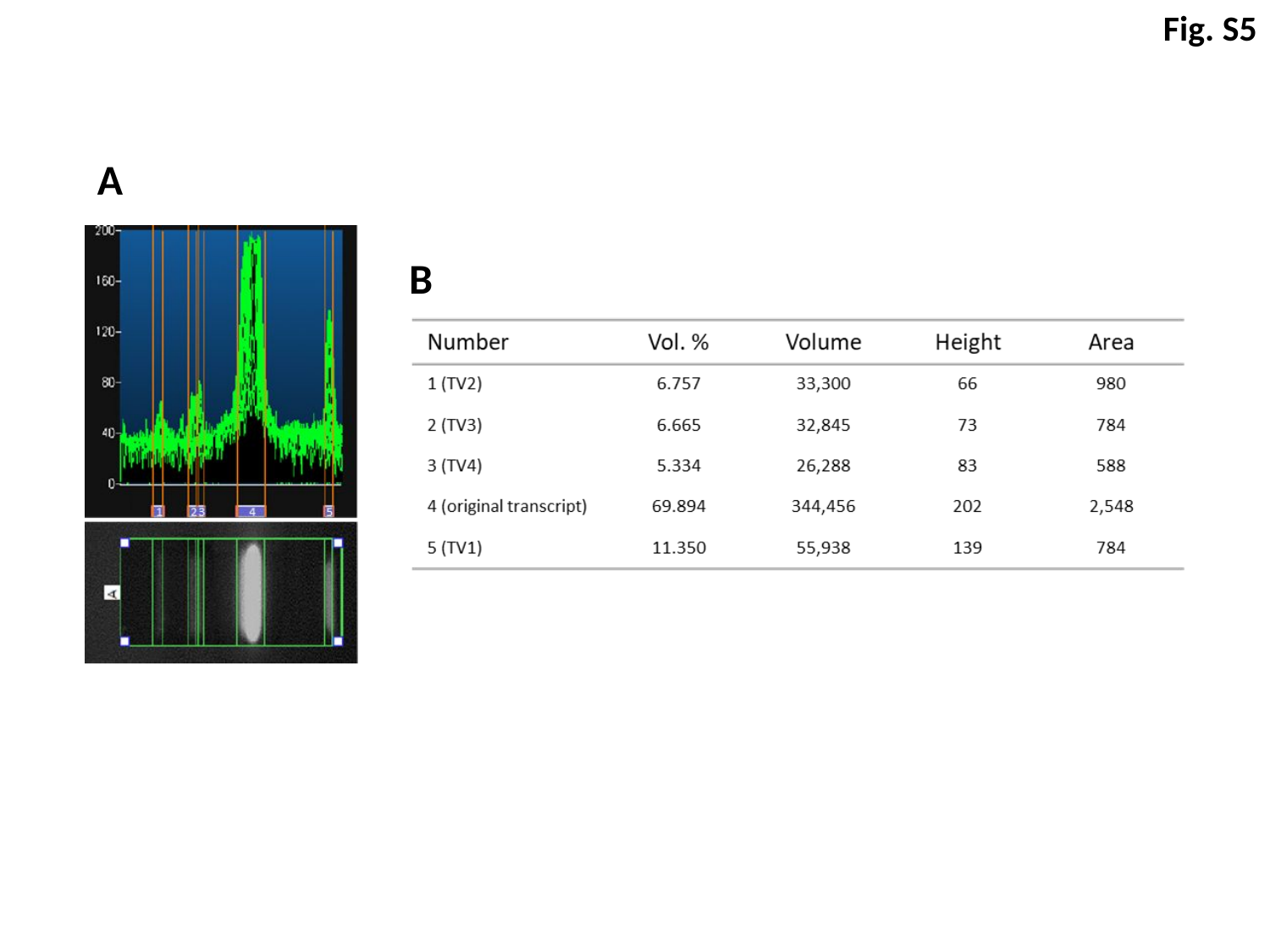

Fig. S5
A
B
